# Infection with *Scedosporium apiospermum* and *S*. *prolificans*, Australia

**DOI:** 10.3201/eid1308.060576

**Published:** 2007-08

**Authors:** Louise Cooley, Denis Spelman, Karin Thursky, Monica Slavin

**Affiliations:** * Alfred Hospital, Prahran, Victoria, Australia; † Royal Hobart Hospital, Hobart, Tasmania, Australia; ‡ Royal Melbourne Hospital, Parkville, Victoria, Australia

**Keywords:** Scedosporium prolificans, Scedosporium apiospermum, Australia, immunocompetence, immunocompromised host, fungemia, respiratory tract colonization, stem cell transplantation, organ transplantation, research

## Abstract

*Scedosporium apiospermum* and *S. prolificans* are fungi of increasing clinical importance, particularly in persons with underlying diseases. We reviewed the records of 59 patients in Australia from whom *Scedosporium* spp. were isolated from June 30, 1997, through December 31, 2003. *S. apiospermum* was isolated predominantly from the respiratory tracts of 28 of 31 patients with underlying lung diseases and resulted in 2 infections and 1 death. The annual number of *S. apiospermum* isolates remained constant. *S. prolificans* was isolated from 28 patients only after November 1999. Eight patients with acute myeloid leukemia or hematopoietic stem cell transplants had invasive infection; 4 had fungemia and 6 died from infection. *S. prolificans* caused locally invasive infection in 2 immunocompetent patients and was found in the respiratory tract of 18 patients with underlying respiratory disease but did not cause fungemia or deaths in these patients. *Scedosporium* spp. showed distinct clinical and epidemiologic features.

## Abstract 2

*S. prolificans* has become a major pathogen in immunocompromised patients.

*Scedosporium apiospermum* and *S*. *prolificans* are saprophytic molds with a worldwide distribution. *S. apiospermum*, the anamorph of *Pseudallescheria boydii*, was described more than a century ago as a cause of Madura foot and subsequently mycetoma and otitis externa. Recently, it has been isolated from patients with chronic lung disease, particularly cystic fibrosis (*1*,*2*), where the spectrum of infection ranges from colonization to disseminated infection. *S. prolificans* was first described as a human pathogen in 1984 (*3*). *Scedosporium* spp. cause a broad spectrum of diseases, including soft tissue infections (*4*–*6*), septic arthritis (*7*), osteomyelitis (*8*,*9*), ophthalmic infections (*10*–*12*), sinusitis (*13*), pneumonia (*14*,*15*), meningitis and brain abscesses (*16*,*17*), endocarditis (*18*), and disseminated infection (*8*,*18*–*21*). Patients at risk include those immunocompromised because of advanced HIV infection (*6*,*18*,*20*), immunosuppressive therapy and neutropenia (*6*,*18*–*21*), or intravenous drug use (*6*,*20*).

In the final months of 1999 during construction work, an increased number of *Scedosporium* spp. isolates and *S*. *prolificans* were noted at Alfred Hospital, a university hospital in Prahran, Victoria, Australia, that provides statewide trauma, burn, cystic fibrosis, heart and lung transplant, and HIV services. The records of all patients from whom *Scedosprium* spp. were isolated from June 30, 1997, through December 31, 2003, were reviewed to describe the epidemiology, clinical features, and outcomes of these infections.

## Methods

### Data Collection

Records of all patients with *Scedosporium* spp. cultured between June 30, 1997, and December 31, 2003, were reviewed retrospectively, and demographics, primary illness, antifungal therapy, and presence of other pathogens, and outcomes were recorded. Immunocompromised patients were defined as those with impairment of either or both natural and specific immunity to infection (*22*). Those with localized impaired host defenses caused by underlying diseases such as cystic fibrosis were classified as immunocompetent. Invasive infection was defined as a tissue biopsy specimen with hyphae plus culture of the organism (*23*). Disseminated infection was defined as positive blood cultures or infection at >2 noncontiguous sites within 7 days. Neutropenia was defined as an absolute neutrophil count <0.5 cells/mm^3^. Descriptive statistics and odds ratios were calculated by using STATA release 8.0 (Stata Corporation, College Station, TX, USA.)

### Laboratory Methods and Epidemiology

*Scedosporium* spp. were identified after culture onto horse blood agar and Sabaroud dextrose agar (SDA) and incubation at 30°C and 37°C. Colonies were rapid-growing, gray-white, and downy, with a gray-black reverse side. Species were differentiated by microscopic morphology (*24*). Susceptibility testing was performed according to Clinical and Laboratory Standards Institute methods (*25*) and synergy studies (2-dimensional 2-agent microdilution checkerboard method) (*26*). Synergy was a summation of the fractional inhibitory concentration <0.500.

For comparison of temporal patterns of mold isolates, the number of persons with *Scedosporium* spp. during the 3 years subsequent to this review (2004–2006) and *Aspergillus* spp. for 1999–2005 were extracted from laboratory databases. Rates of detection were expressed as per 1,000 separations and per 100,000 inpatient-patient days.

### Environmental Sampling

From March 2001 through July 2002, environmental samples were collected from hospital public access areas within and adjacent to the construction site on 7 separate occasions. Additional sampling was conducted in ward corridors, nursing stations, patient rooms, and the patient car park. Air sampling was conducted with a portable high-volume air sampler (MAS 100; Merck, Darmstadt, Germany). The volume of air sampled at each site was 1,000 L/10 min (1 m^3^). Settle plates were placed in ward corridors and patient rooms for 60 min of exposure. Dust was collected from horizontal surfaces with sterile swabs moistened with sterile saline. Dust and soil specimens were directly placed on standard SDA and selective SDA containing amphotericin B and incubated at 27°C.

## Results

From June 1997 through December 2003, a total of 59 isolates of *Scedosporium* spp. were cultured from 56 patients. *S*. *apiospermum* was isolated from 31 patients, and *S*. *prolificans* was isolated from 28 patients. Both species were isolated from 3 patients at separate times (12 days, 13 months, and 27 months apart, respectively). Demographic information, coinfections, and outcomes of the patients are shown according to the species isolated (Table 1). During the period of this review, an average of 28 allogeneic stem cell, 35 lung, 27 heart, and 15 renal transplants were performed each year.

**Table 1:** Characteristics and outcomes of patients infected with *Scedosporium* spp., Australia, 1997–2003

Characteristic	*S. apiospermum*	*S. prolificans*
No. patients	31	28
Patient demographics		
Mean age (range), y	40	42
Sex (M:F)	19:12	16:12
Immunocompromised (%)	21 (68)	14 (50)
Stem cell transplant	0	6
Hematologic malignancy	0	2
Lung and/or heart transplant	13	6
HIV infection, cancer, immunosuppression	8	0
Immunocompetent (%)	10 (32)	14 (50)
Cystic fibrosis	5	6
Airways disease*	4	3
Sinusitis	1	3
Other condition†	0	2
Specimen type		
Respiratory tract (%)	27 (68)	20 (71)
Tissue	3	6
Blood	0	4
Other‡	2	1
Additional microorganisms identified (%)	16 (42)	14 (50)
Molds§	9	10
Bacteria¶	9	8
Outcome at 1 mo		
Invasive infection (%)#	2 (6)	10 (36)
Died at 1 mo (%)**	6 (19)	5 (18)
Died of scedosporiosis (%)††	1 (3)	5 (18)
Attributable mortality rate of invasive disease (%)‡‡	1/2 (50)	5/10 (50)

*Bronchiectasis, asthma, and chronic obstructive pulmonary disease. †Osteoarthritis and trauma. ‡Ear swab, central catheter tip, and synovial fluid. §*Aspergillus* spp, *Rhizopus orrhyzae*, and *Paecilomyces* sp. ¶Bacteria not found in normal flora included *Pseudomonas aeruginosa*, *Stenotrophomonas maltophilia*, *Staphylococcus aureus*, *Nocardia* sp., and *Mycobacterium avium* complex. #Odds ratio (OR) 6.64, 95% confidence interval (CI) 1.62–27.12, p = 0.001. **OR 1.11, 95% CI 0.40–3.04, p = 0.84. ††OR 5.53, 95% CI 0.68–44.54, p = 0.06. ‡‡OR 1.0, 95% CI 0.21–4.56, p = 1.0.

### *S*. *apiospermum*

*S*. *apiospermum* was isolated from 32 specimens collected from 31 patients. Twenty-nine isolates were from the respiratory tract; sputum (n = 22), bronchoalveolar lavage (BAL) (n = 5), sinus (n = 1), and lung tissue (n = 1). The remaining 3 isolates were from brain tissue, a central venous catheter tip, and an ear swab, respectively. No blood cultures were positive for *S*. *apiospermum*. *S*. *apiospermum* was isolated concurrently with *Aspergillus* spp. from the respiratory tracts of 9 (29%) of the 31 patients.

Twenty-one (68%) of the 31 patients were immunocompromised. Thirteen had undergone solid organ transplantation (11 had lung transplants and 2 had heart transplants), 4 had malignancies (3 had metastatic cancer and 1 had chronic lymphocytic leukemia), 3 had advanced HIV infection, and 1 was receiving immunosuppressive therapy for rheumatoid arthritis. All 10 immunocompetent patients had chronic respiratory tract disease; cystic fibrosis (n = 5), bronchiectasis (n = 4) and chronic mastoiditis (n = 1).

Three patients were lost to follow-up after isolation of *S*. *apiospermum.* The remaining 28 patients were followed up for a total of 981 months. The mean duration of follow-up was 35 months (median 16 months, range 1–96 months). Two (6%) of the 31 patients had invasive infections. The first patient was a woman who received a lung transplant 5 years earlier and was previously colonized with *Aspergillus* spp. but not *S*. *apiospermum*. She had pulmonary and cerebral lesions and was treated with amphotericin B deoxycholate and itraconazole for presumed aspergillosis. *S*. *apiospermum* was isolated from postmortem lung and brain tissue. The second patient was a man who had advanced HIV infection with fungal sinusitis on biopsy. He was treated with voriconazole and surgery but died 3 months later from infection with cytomegalovirus. There were 6 deaths within 1 month of isolation of *S*. *apiospermum*. All deaths occurred in immunocompromised patients but only 1 was directly attributable to *S*. *apiospermum*. The other deaths resulted from the underlying condition.

Four patients with chronic lung disease were receiving itraconazole for treatment of *Aspergillus* spp. infection at the time *S*. *apiospermum* was isolated. Three of these patients died of respiratory failure 7, 9, and 16 months, respectively, after isolation of *S*. *apiospermum*, and 1 was alive when last seen 96 months after fungal isolation. Seven patients received antifungal therapy after isolation of *S*. *apiospermum* from respiratory tract samples (4 received voriconazole and 3 received itraconazole). These 7 patients had HIV infection and sinusitis (n = 2), had undergone lung transplantation (n = 3) or had bronchiectasis or cystic fibrosis (n = 2). Both patients with HIV infection had sinusitis and died within 7 months of complications of HIV. Of the other 5 patients, 4 who received azole therapy remained well without invasive infection at 32, 41, 48, and 88 months, respectively, of follow-up. The remaining treated patient died 15 months later; death was not attributed to fungal infection. The median duration of follow-up of those treated with azoles at the time of fungal isolation or subsequent to isolation was 16 months (range 3–96 months); *S*. *apiospermum* was not subsequently detected in these patients. The median duration of follow-up for patients receiving no treatment after fungal isolation was 19 months (range 1–84 months); *S*. *apiospermum* was isolated from 4 of these patients 1, 18, 30, and 36 months, respectively, after initial fungal isolation.

### *S*. *prolificans*

*S*. *prolificans* was isolated from 46 specimens obtained from 28 patients. Fourteen (50%) of the 28 patients were immunocompetent. Most (12/14, [86%]) specimens from immunocompetent patients were from the respiratory tract; cystic fibrosis (n = 6), chronic airway disease (n = 3), nasal discharge and sinus aspirates with chronic sinusitis (n = 3). Patients with cystic fibrosis or airway disease were not considered to have invasive disease and none received antifungal therapy. All 3 patients with chronic sinusitis were treated with surgery, and 2 received itraconazole. One isolate was from knee cartilage of a patient with hemophilia, osteoarthritis, and a knee replacement; the patient underwent surgery and received itraconazole. *S*. *prolificans* was also isolated from a skin-biopsy specimen from a patient with multiple-trauma and cellulitis who was treated only with surgery. There were no cases of disseminated infection or deaths resulting from *S*. *prolificans* in these immunocompetent patients.

*S*. *prolificans* was isolated from 14 immunocompromised patients. Immunodeficiency was caused by lung transplantation (n = 6), hematopoietic stem cell transplantation (HSCT) (n = 6), acute myeloid leukemia (AML), and myelodysplastic syndrome (MDS). *S*. *prolificans* was isolated from BAL of 6 lung transplant recipients. One patient was receiving itraconazole for *Aspergillus* spp. infection, 3 were receiving voriconazole, and 2 were receiving itraconazole and voriconazole with terbinafine. Invasive disease did not develop in any of the 4 lung transplant recipients who received antifungal treatment and none died. Two lung transplant recipients did not receive antifungal treatment. One of these patients was lost to follow-up and 1 survived >25 months without developing invasive infection, although *S*. *prolificans* was isolated again 1 year later.

*S*. *prolificans* was isolated from August 2000 through September 2002 from 6 patients undergoing allogeneic HSCT and from 2 patients with hematologic malignancy. Four of the 6 HSCT recipients had positive blood cultures, and 4 recipients had skin lesions (multiple, nodular). Findings for 8 patients in whom invasive disease developed are summarized in Table 2.

**Table 2:** Characteristics of 8 patients with invasive disease and *Scedospoeium prolificans* infections, Australia, 1997–2003*

Patient no.	Primary disease	Transplant type	Neutropenia within 30 d	GVHD	Date of first isolate	Days post transplant	Initial symptom	Site of isolates	Outcome following diagnosis
1	ALL	Allogeneic HSCT	Yes	Chronic extensive	Aug 2000	>400	Knee effusion	Blood, synovium, cartilage, prostate	Died d 21
2	AML	Allogeneic HSCT	No	Chronic extensive	Apr 2001	>500	Pulmonary infiltrate	Blood, sputum, BAL, lung	Died d 5
3	MM	Allogeneic HSCT	Yes	No	Nov 2000	28	Ethmoid sinus infiltrate	Ethmoid sinus, vertebral disc, mycotic aneurysm	Alive d 500
4	NHL	Allogeneic HSCT	Yes	No	Dec 2000	10	Neutropenic sepsis	Blood	Died before diagnosis
5	AML	Allogeneic HSCT	Yes	No	Mar 2003	10	Neutropenic sepsis	Blood, sputum, BAL, lung, skin	Died d 1
6	MM	Allogeneic HSCT	Yes	Chronic extensive	Nov 2002	120	Pneumonia	Sputum	Died d 1
7	MDS	NA	No	NA	Nov 2003	NA	Maxillary sinus infiltrate	Sputum, maxillary sinus, pericardium, myocardium, kidney, skin, lung	Died d 14
8	AML	NA	Yes	NA	May 2002	NA	Catheter-related sepsis	Chest wall, Hickman catheter	Alive d 500

*GVHD, graft versus host disease; ALL, acute lymphoblastic leukemia; HSCT, hematopoietic stem cell transplantation; AML, acute myeloid leukemia; BAL, bronchoalveolar lavage; MM, multiple myeloma; NHL, non-Hodgkin lymphoma; MDS, myelodysplastic syndrome; NA, not applicable because patients did not undergo stem cell transplantation.

Patients 1, 2, and 6 had HSCT complicated by chronic extensive refractory graft versus host disease (GVHD). All 3 died of invasive infection despite treatment with itraconazole or voriconazole and terbinafine; 2 had positive blood cultures. Infections developed in patients 3, 4, and 5 during neutropenia after HSCT. Two of them had positive blood cultures, and both died. Patient 4 received no antifungal treatment, and patient 5 died despite replacement of prophylactic itraconazole with empiric amphotericin B. Patient 3, described elsewhere (*8*), survived after treatment with voriconazole, terbinafine, surgery, and neutrophil recovery. Patient 7 had MDS and preceding idiopathic CD4-cell lymphocytopenia. *S*. *prolificans* was isolated from sputum, but not BAL, and this patient was not treated. One month later, *S*. *prolificans* sinusitis was diagnosed. Despite surgery and treatment with itraconazole, followed by voriconazole and terbinafine, the patient died. Patient 8, a woman, was neutropenic after remission-induction chemotherapy for AML. A swab from a Hickman catheter exit site was positive for *S*. *prolificans*. Subsequently, chest wall cellulitis, deep soft tissue infection, and multiple skin nodules developed. She was treated with surgery, voriconazole, and terbinafine. Her neutropenia resolved and she recovered.

After isolation of *S*. *prolificans*, 5 of 28 patients were lost to follow-up. The remaining 23 were followed up for a total of 517 months. Mean length of follow-up was 22.5 months (median 9 months, range 1–68 months). Invasive infection occurred in 10 (36%) of 28 infected with *S*. *prolificans* compared with 2 (6%) of 31 infected with *S*. *apiospermum*. Deaths caused by scedosporiosis occurred in 5 (18%) of 28 infected with *S*. *prolificans* and 1 (3%) of 31 infected with *S*. *apiospermum*. All deaths occurred in immunocompromised patients; 6 (43%) of 14 infected with *S*. *prolificans* and 1 (5%) of 21 infected with *S*. *apiospermum* (odds ratio 6.0, 95% confidence interval 0.78–45.62, p = 0.05).

### Drug-Susceptibility Testing

Drug-susceptibility testing was performed on 7 *S*. *prolificans* isolates. MICs (mg/L) were >16 for amphotericin B, >64 for fluconazole, >8 for itraconazole, >64 for 5-fluorocytosine, and >16 for ketoconazole, 2–8 for voriconazole, and 1–4 for terbinafine. Terbinafine and itraconazole were synergistic for 3 of 6 isolates tested, and terbinafine and voriconazole were synergistic for 5 of 7 isolates tested. Four *S*. *apiospermum* isolates tested had MICs of 0.5–1 mg/L for itraconazole and 0.5 mg/L for voriconazole.

### Epidemiology of *Scedosporium* spp. and *Aspergillus* spp.

Throughout the study, *S*. *apiospermum* was isolated at a constant rate (Figure) of 3–6 isolates per year with no seasonal clustering. In contrast, *S*. *prolificans* was first detected in December 1999, and thereafter ≈8 isolates were identified annually. Detection of *S*. *prolificans* continued in 2004 with 8 isolates but decreased to 6 in 2005 and 4 in 2006. Two clusters of infection occurred in autumn 2001 and 2003 during periods of hospital building that required deep excavation. Air, dust, and soil sampling did not detect an environmental fungal source. *Aspergillus* spp. were detected during 1999–2005 (mean 191 persons per year, range 153–230). Detection rates for *Scedosporium* spp. and *Aspergillus* spp. for 2003–2005 were 0.1–0.23/1,000 separations and 2–3/100,000 inpatient-patient days and 2.4–3.0/1,000 separations and 53–75/100,000 inpatient-patient days, respectively.

**Figure:**
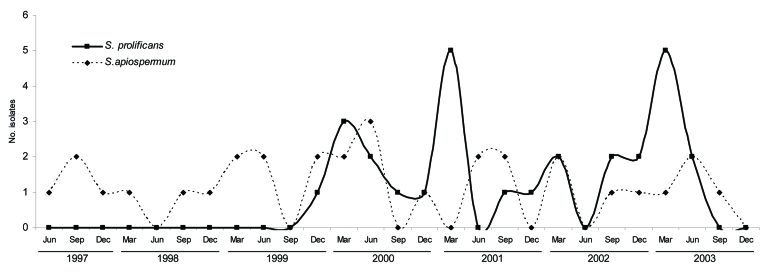
Epidemiologic curve of isolation of *Scedosporium* spp. isolation, Australia, June 1997–December 2003. *S. prolificans* was first identified in December 1999 and had 2 peaks that coincided with construction work. *S. apiospermum* was isolated at a constant rate of 1–2 times per 3-month period.

## Discussion

This study describes the epidemiology of *Scedosporium* infection in a cohort of contemporaneous patients at a university hospital. The single-center approach, with cases identified by laboratory detection, allows capture of the full spectrum of infection from colonization to invasive infection. *Scedosporium* spp. were detected in a broad range of patients and clinical settings. However, there were distinct differences in epidemiology, clinical manifestations, antifungal susceptibility patterns, and outcomes between *S*. *prolificans* and *S*. *apiospermum*.

Previous reviews have focused on invasive cases reported (*10*,*27*,*28*), but this approach is limited by both selection and publication bias (*29*) and does not describe the natural history of infection or the prevalence of these infections. Our series enables the annual incidence of cases, ratio of colonized to infected patients, and natural history of colonization to be determined for each species. We showed that invasive infections accounted for only 6% of *S*. *apiospermum* isolates and for 46% of *S*. *prolificans* isolates, and that isolation of *Aspergillus* spp. was 20–30 times more frequent than that of *Scedosporium* spp.

*S*. *apiospermum and S*. *prolificans* are colonizers of abnormal airways caused by bronchiectasis, cystic fibrosis, chronic obstructive pulmonary disease, or lung transplantation (*1*,*2*,*6*,*18*,*30*,*31*). *S*. *apiospermum* was an airway colonizer in <10% of patients with cystic fibrosis in France and Australia (*1*,*2*), and similar proportions of lung transplant recipients were colonized with 1 or both species at 1 center in Australia (*31*). However, there are few reports that *S*. *prolificans* colonized patients with lung disease (*6*,*32*). In the present study, *S*. *prolificans* was identified as an airway colonizer only after December 1999. The onset of invasive infections in HSCT and neutropenic patients occurred in August 2000. *S. prolificans* was first detected at the M.D. Anderson Cancer Center in Houston, Texas, after 2001 (*19*). Emergence of *S*. *prolificans* may be related to an environmental source that was not identified or selection pressure caused by changes in antifungal prophylaxis practices (*33*). This finding did not appear to be the explanation in our patients because fluconazole remains standard prophylaxis for neutropenia, and itraconazole was used in only 1 of 8 patients. Other possible explanations include use of more aggressive chemotherapy regimens in patients with acute leukemia and the advent of nonmyeloablative allografts, which change characteristics of patients selected for transplants. The reason for persistence of this organism over several years after its initial appearance is also unclear but has also been observed by others (*19*).

In patients with respiratory tract disease, both *Scedosporium* spp. were isolated in comparable numbers. However, the only invasive infection in this diverse group was disseminated *S*. *apiospermum* 5 years after lung transplantion. The outcome of 19 patients who had undergone lung or heart lung transplantation and were colonized with this fungus was comparable to the 17 patients in the immunocompetent group with airways disease caused by cystic fibrosis, bronchiectasis, or chronic obstructive pulmonary disease. As in other reports, other opportunistic pathogens, especially *Aspergillus* spp. (present in one third), were commonly cultured simultaneously (*1*,*18*,*30*,*31*). In lung transplant recipients, *Scedosporium* spp. in BAL was associated with advanced bronchiolitis obliterans and airway stenosis (*31*), which emphasizes the difficulty of interpreting the role of *Scedosporium* spp. in this group. Whether colonization follows airway damage, immunosuppression, or antifungal therapy to treat *Aspergillus* spp. colonization or is itself an ongoing cause of airway damage such as bronchiolitis obliterans is unknown. In our study group, most patients with respiratory tract colonization had not received antifungal therapy. Although antifungal treatment was used in ≈50% of patients with *S*. *apiospermum* airway colonization, there appeared to be no survival advantage in patients treated; our study was not a randomized comparison. With *S*. *prolificans* respiratory tract colonization, too few patients were treated with antifungal drugs for valid conclusions to be made. There was no reduction in survival in 5 untreated patients with isolates of either *S*. *apiospermum* or *S*. *prolificans*. The role of *Scedosporium* spp. respiratory tract colonization requires further evaluation.

Allogeneic HSCT and AML/MDS were the only settings in which *S*. *prolificans* was more common than *S*. *apiospermum.* There were no *S*. *apiospermum* infections in these patients, although in at 1 US cancer center, *S*. *apiospermum* infections were more common (*19*). This may reflect different geographic distributions of *Scedosporium* spp. In our study, *S*. *prolificans* caused illness with high mortality rates in HSCT and leukemia patients. In HSCT recipients, *S*. *prolificans* infections occurred equally in those with neutropenia (early after transplant) and GVHD (later after transplant). Thus, host factors unique to AML and HSCT, such as neutropenia, GVHD, associated macrophage dysfunction, and environmental exposure, may play a role in the propensity for invasive infection with *S*. *prolificans* in this group.

As in previous reports (*10*,*32*), neutropenic patients had sepsis, positive blood cultures, and overwhelming infection (often associated with disseminated rash). In nonneutropenic patients, infection was more likely to be localized initially in lungs, joints, or sinuses. The ability of *S*. *prolificans* to grow in blood cultures in AML and HSCT patients is well recognized (*10*,*34*), and this diagnosis, or infection with *Fusarium* spp., should be considered when a mold is cultured from blood. In our study, *S*. *prolificans* was the only species to grow in blood culture, although others have reported *S*. *apiospermum*, albeit, less frequently (*10*,*18*,*19*). Although colonization with *S*. *prolificans* without progression to disease was observed in patients with respiratory disease and after lung transplantation, this was not observed in 3 patients with AML/MDS or HSCT. A nonsterile site swab or sputum culture yielding *S*. *prolificans* was soon followed by a diagnosis of invasive infection, which indicated that in these patients a culture result, even from a nonsterile site, should be viewed seriously.

The clinical findings of *S*. *prolificans* in our patients suggest that this fungus is more invasive than *S*. *apiospermum*, as has been found in mice (*35*). In addition to disseminated infection in AML patients and HSCT recipients, *S*. *prolifican*s also caused locally invasive disease in patients who were not immunocompromised, such as those with posttrauma cellulitis, septic arthritis in a damaged joint, and sinus disease. One proposed mechanism for virulence of *S*. *prolificans* is melanin in the cell wall (*27*).

Antifungal therapy is problematic with *S*. *prolificans* with high MICs for amphotericin B, echinocandins, and azoles. This finding has stimulated interest in combinations of voriconazole and terbinafine (*26*), voriconazole and echinocandin (*36*), or polyene and echinocandin (*36*). However, these infections are rare and experience is limited to case reports (*8*,*9*,*36*). In vitro treatment with interferon-γ and granulocyte-macrophage–colony-stimulating factor has improved neutrophil function against *S*. *prolificans* hyphae (*37*). Surgery appears to be an important factor in survival in our cases, as well as other series (*10*). Although surgery and antifungal therapy were successful in 2 neutropenic patients with invasive *S*. *prolificans* infections, these patients also had concomitant neutrophil recovery that could explain their survival.

Much needs to be learned about the epidemiology, transmission, pathogenesis, and environmental niche of *Scedosporium* spp., especially *S*. *prolificans*. A source for emergence of *S*. *prolificans* at our hospital was investigated, but none was identified. Our inability to detect the source of emergent *S*. *prolificans* was puzzling, although exposure and colonization may have occurred by the time the first case was seen. Cultures may have been obtained too late to identify environmental contamination, although changes in immunosuppression in the population at risk may also have contributed. *S*. *prolificans* appeared first as a colonizer of abnormal airways 8 months before the first invasive infection in an HSCT recipient. Shifts in the epidemiology of colonizing organisms in this patient group during hospital construction may be worthy of closer attention and surveillance. Molecular typing methods such as PCR (*38*) and inter-simple sequence repeat PCR fingerprinting (*39*) have distinguished genotypes from outbreaks and different geographic regions. Recently, *S*. *apiospermum* complex has been shown to include several individual species indistinguishable morphologically (*40*). These methods will be used for further investigation of our isolates as part of a larger Australian surveillance study.

In conclusion, *S*. *prolificans* and *S*. *apiospermum* are pathogenic fungi that demonstrate distinct clinical features dependent on the immune function of the host and the type of species isolated. They are usually found in patients with underlying disease, although occasionally after trauma. *S*. *prolificans* emerged as a major pathogen in allogeneic HSCT recipients and as a colonizer of patients with underlying lung disease. Madura foot is now rare and was not observed in this series. The high attributable mortality rate of invasive infection with both *Scedosporium* spp., limitations of antifungal therapy, necessity for aggressive and deforming surgery to treat infections with *S*. *prolificans*, and uncertainty over the role of airway colonization emphasize the need to better understand the epidemiology and pathogenesis of this infection.
